# Pregnant adolescents and nurses perspectives on accessibility and utilization of maternal and child health information in Ohangwena Region, Namibia

**DOI:** 10.1186/s12884-022-04619-w

**Published:** 2022-04-05

**Authors:** Joyce T. Shatilwe, Khumbulani Hlongwana, Tivani P. Mashamba-Thompson

**Affiliations:** 1 Discipline of Public Health Medicine, School of Nursing and Public Health, University of KwaZulu-Natal, Durban, South Africa; 2 Faculty of Health Sciences, University of Pretoria, Pretoria, South Africa

**Keywords:** Accessibility, Utilization, Pregnancy, Adolescent girls, Maternal and child health information

## Abstract

**Background:**

Adolescent reproductive health is still a challenge in Low and Middle Income Come Countries (LMICs). However, the reasons for the inability of most pregnant adolescent girls to access and utilize maternal and child health information (MCHI) are not well-documented. This is despite the policy guidelines promoting the provision of this necessary information to pregnant adolescents in order to prepare them for delivery. This provision is one of the strategies envisaged to improve their attendance of ANC visits and their maternal and child health.

**Method:**

Data were generated from 12 adolescent pregnant girls aged 15 to 19 years and eight nurses from four different health centres in the Ohangwena Region of Namibia, using semi-structured in-depth interviews. The study was conducted over the period of three months (December 2018 to March 2019). The data were grouped into clusters aided by NVivo computer software version 12. Data were organized and condensed in small units, prior to being coded, categorized, and finally grouped into main themes and sub-themes.

**Results:**

Results revealed that long travel hours to reach the nearest clinics was amongst the leading challenges affecting accessibility and utilization of MCHI for pregnant adolescent girls. This was exacerbated by poor support with transport fare, poor road infrastructure and non-availability of transport, and these factors were key barriers to accessibility and utilization of clinic services. Other barriers pertained to the family dynamics, such as disclosing the pregnancy to the family members prior to commencing antenatal care (ANC) visits and harsh treatment from family members after the disclosure.

**Conclusion:**

The pregnant adolescent girls were concerned about their inability to access and utilize MCHI, thereby making them susceptible to maternal complications. Health educational interventions should prioritize both the adolescent girls and their families for proper support, especially since the reactions of families on the pregnancy of their adolescent girls often negatively affect accessibility and utilization of maternal and child health services. Moreover, further research on adolescents' needs during pregnancy should be expanded to include their parents, in order to better inform policymakers.

**Supplementary Information:**

The online version contains supplementary material available at 10.1186/s12884-022-04619-w.

## Background

Approximately 99% of the world's maternal deaths occur in resource-limited settings [1]. Sub-Saharan Africa (SSA) alone accounts for two-thirds (66%) of deaths [1], with a number of 2017/2018 publications reporting maternal deaths of 546 per 100 000 live births in the region [2, 3]. The lifetime risk of death for women in high-income countries (HICs) is as low as 1 in 2400, compared to 1 in 180 in low-income countries (LICs) [1, 4]. In September 2000, world leaders signed the United Nations Millennium Development Declaration, which committed countries to reducing child mortality by two-thirds (MDG 4), reducing the maternal mortality ratio by three quarters (MDG 5A), and achieving universal access to reproductive health (MDG 5B) between 1990 and 2015 [5]. There are now new global commitments, such as the new Global Strategy for Women's, Children's and Adolescents' Health, ending the AIDS epidemic by 2030 and the sustainable development priorities. Namibia was a signatory country to the United Nations declaration [5]. While Namibia, as an upper-middle-income country in SSA, has achieved the target for births to be attended to by skilled health providers, it fell short of meeting the target for the expired Millennium Development Goals (MDGs), which aimed to reduce maternal deaths to 56 for every 100 000 live births by 2015 [5].

In Namibia, adolescent pregnancy has increased from 15% in 2006 to 18.6% in 2013 [6]. The number of adolescent pregnancies has remained high in the country, with some areas recording up to 38.9% adolescent pregnancies [5]. Adolescent girls are more likely to experience pregnancy-related complications due to obstructed labour and eclampsia, thereby increasing their risk of death [7]. Children born to adolescents are also more likely to have a low birth weight, ill-health, stunting, and other poor nutritional outcomes [1]. Most of these complications are preventable if pregnant women are prepared and they timeously access and utilize necessary MCHI for childbirth [4, 8]. Information and education are basic sexual and reproductive health rights; hence, increasing access to information on reproductive rights gives people choices and a sense of entitlement to quality services [5]. The implementation of the above mentioned new global targets and goals is anticipated to improve the maternal healthcare of many women and children, especially in resource-limited settings.

There are several challenges that hinder the achievements of global targets to improve maternal and child healthcare, and these include accessibility and utilization of MCHI [9, 10]. The literature has shown that continued poor and unequal access to skilled maternal and child healthcare services remains a major challenge, not only in Namibia, but in the majority of resource-limited settings [10–13]. The challenges further contribute to poor accessibility and utilization of MCHI, since adolescent girls mostly get information when they present at health facilities for maternal health services [8]. In most Low-Middle-Income countries, there are a number of challenges that hinder the access and utilization of this health information, and these include distance to the nearest health facilities, delay in decision-making, financial constraints, and the attitudes of health professionals [8, 14].

There are multiple potential explanatory factors for the under-utilization of maternal and child care services, such as young maternal age; religion; poor education; unskilled occupation; poverty; low caste; parity (higher birth order were less likely to access antenatal services); lack of autonomy; poor familial support; lack of access to transport; and the high cost of care, and when it is accessed, the poor quality [15–17]. While some studies have also highlighted a wide number of factors that influence the utilization of MCHI, including education, distance to the nearest health facilities, lack of network coverage, family members, lack of involvement in community engagement activities, lack of youth-friendly initiatives, socio-economic and cultural factors, literacy levels, economic status, lack of health outreach programmes and language [18–20], the effect of information and access to it and how it influences maternal health remains unknown, especially in marginalized and rural communities.

Namibia is committed to reducing maternal mortality, and this is evidenced by the implementation of life-saving skills training of trainers, routine maternal death reviews, improved infrastructure, strengthened adolescent sexual and reproductive health and rights, and improved prevention of mother-to-child transmission (PMTCT) strategies [15]. However, these initiatives need to be supported by local data from appropriately designed studies.

We, therefore, explored the user-provider perspectives towards the accessibility and utilization of MCHI by pregnant adolescents in the Ohangwena Region in Namibia. The study was underpinned by the Theory of change, which asserts that change is a rigorous yet participatory process whereby groups and stakeholders in a planning process articulate their long-term goals and identify the conditions they believe have to unfold for those goals to be met [21]. These conditions are modeled as desired outcomes, arranged graphically in a causal framework. It covers the following elements: basic elements of needs (the initial problem being addressed), inputs (resources), outputs (intended activities) and outputs (desired changes for service users) [22].

The main aim of the study was to explore the perspectives for pregnant adolescents and nurses on accessing and utilization of maternal and child health information in Ohangwena Region, Namibia. It is anticipated that the results of this study will be useful to the Government of the Republic of Namibia, the community, and non-governmental organizations in improving the accessibility and utilization of maternal and health information by adolescent girls, in order to improve maternal health outcomes among this population group.

## Methods

### Research paradigm

This study was guided by the interpretive paradigm due to its ability to explore the phenomenon from the perspectives of the service users (adolescent girls) and service providers (nurses) [23, 24]. By applying an interpretive paradigm, the researchers had a chance to view the world through the lenses and experiences of those participating in the provision and use of MCHI in their region.

### Study design

An exploratory qualitative design was applied to explore the experiences of service users and service providers pertaining to the accessibility and utilization of MCHI during pregnancy in the Ohangwena Region in Namibia.

### Study setting and study population

The study was conducted in the Ohangwena Region, which is one of the 14 political regions in Namibia. It shares international boundaries with Angola to the north and regional boundaries with the Kavango region to the east, Omusati region to the west, and Oshana and Oshikoto regions to the south. It is the second-highest populated region in Namibia with a population of 274 650 [25]. Ohangwena is a rural region and one of the most poverty-stricken areas in the country [26]. In 2017, Ohangwena was rated as one of the regions with the highest prevalence of teenage pregnancy [26]. The major diseases in the region are pneumonia, malaria, diarrhoea, and HIV/AIDS [26]. The total population of pregnant women between the target ages of 15 and 19 was 693. The total number of registered nurses and enrolled nurses and midwives working at the selected health centres and clinics was 57.

### Sample size and sampling strategy

The study participants included twelve (12) pregnant adolescents from different villages across Ohangwena Region and eight (8) Nurses from two different health professional categories (registered nurse and enrolled nurse and midwifery) who were working in health centres and clinics, Ongha Health Centre, Odibo Health Centre, Engela Clinic and Eenhana Clinic in Ohangwena Region.

A purposive sampling strategy was applied to identify 12 participants out of the 98 potential participants from four purposively selected sites. The identification of potential participants was based on their professional categories: one nurse dealing with antenatal consultations per professional category per site (enrolled Nurse and registered nurse), culminating in the selection of two nurses per site. Furthermore, these potential participants were considered information-rich, based on their inputs into the quantitative aspects of the study.

Only the adolescents who were pregnant at the time of the data collection, aged 15 to 19 years old, 1–40 gestational weeks pregnant and resident of the Ohangwena Region, were considered for inclusion in the study. Excluded in the study were women below the age of 15 or older than 19, not from the Ohangwena Region, and not pregnant at the time of data collection. For the selection of the nurses, the following inclusion and exclusion criteria were applied: Inclusion criteria: any year of age, working at ANC clinics, registered nurses or enrolled nurse and those from midwifery, male or female; Exclusion criteria: staff members who were not nurses, nurses who were not working at antenatal clinics, nurses who had worked less than one month at an antenatal clinic.

### Data collection

This study was conducted over a three-month period (December 2018 to March 2019). The lead researcher, who had some training on qualitative research, conducted semi-structured in-depth interviews with pregnant adolescents who presented for ANC visits at the four sites and with eight nurses from four different health centres and clinics. An interview guide used to generate data covered the following areas: Reasons for late to start of antenatal care, mode of transport to the clinic, the distance to the nearest clinics, waiting time and challenges affecting access to MCHI. Questions pertained to how pregnant adolescents accessed ANC clinics, including the factors that affected the utilization of services. Questions directed to the nurses pertained to the observed ANC clinics attendance patterns by the pregnant adolescents, including the challenges viewed to affect the service uptake by this age group.

A maximum of four interviews per day were scheduled through telephonic appointments. Each interview lasted for about 30–40 min per participant and no follow-up interviews were conducted. The interviews were conducted in Oshiwambo, since the participants were more comfortable to talk in their own language. The interview venues were determined by the participants to ensure that they were less inconvenienced. A majority of participants preferred to be interviewed at the nearest clinics and some preferred to be interviewed at their place of residence and maternity shelters, as they were closer to their time of giving birth. The interviews for nurses were conducted either at their clinics or health centres. With the participants' permission, note-taking and digital audio-recordings were used to record the data. Code saturation was reached at 10 interviews, as data collection and analysis were conducted iteratively. However, the meaning saturation was reached after interviewing all twelve selected pregnant adolescent girls.

### Data management

Two university students experienced in qualitative research were recruited to transcribe the audio materials verbatim. For quality-check purposes, the lead researcher listened to the audio materials while reading the transcriptions, in order to assure the quality of transcription prior to translating from Oshiwambo to English. Notably, the lead researcher (JS) is competent in both Oshiwambo and English. Furthermore, a Ph.D. graduate from the University of Namibia independently verified the quality of the transcriptions. Data were exported into NVivo computer software version 12 for analysis.

### Data analysis

Thematic analysis techniques were applied to analyze the data, the following process of thematic analysis being followed: Data was condensed in small units, and then the coding process was conducted. The process of categorization of the data followed and, finally, thematic analysis was conducted by classifying the data into sub-themes and main themes. The lead researcher (JS) immersed herself in data through several readings of the transcripts in order to generate concepts. To support the findings, verbatim quotes were extracted from the transcripts.

Five main themes and eighteen sub-themes were identified. The main themes were: Reasons for late starting of ANC, mode of transport to the clinic, the distance to the nearest clinics, waiting time and challenges affecting access to MCHI.

## Results

The pregnant adolescent girls' ages ranged from 15 to19 years and the nurses' ages ranged from 26 to 55 years. All pregnant adolescent girls were from rural settings. The nurses' years of experience ranged from 1 to 30 years. The demographic information for the nurses and pregnant adolescents who participated is presented in Table 1, while Table 2 shows the main themes and sub-themes on which results were based.

**Table 1:** Demographic characteristics of participants who provided their perspectives towards accessibility and utilization of maternal and child health information by pregnant adolescents in the Ohangwena Region, Namibia, (*N* = 12 & *N* = 8)

Participants code (adolescent girls) (*N* = 12)	Age	Distance to the nearest health facility	Level of education (grade)	Education status	Gestational age (months of pregnancy)	Participants code (nurses) (*N* = 8)	Years of experience at ANC clinic (in months)	Age of nurse	Position/Rank
EngCl #21	18	> 5 km	Grade 10	Primary education	7	En#01	60 months	27	Enrolled nurse/midwifery
EngCl #11	18	5 km	Grade 8	Primary education	9	En#02	1 month	26	Enrolled nurse/midwifery
EngCl #08	19	> 5 km	Grade 10	Primary education	8	En#03	13 months	28	Enrolled nurse/midwifery
OngHC #09	15	> 5 km	Grade 9	Primary education	5	En#04	36 months	27	Enrolled nurse/midwifery
OngHC #16	16	3 km	Grade 9	Primary education	6	Reg#01	360 months	55	Registered nurse
ODHC #07	18	5 km	Grade 11	Primary education	7	Reg#02	11 months	26	Registered nurse
EenhCL #17	18	> 5 km	Grade 10	Secondary education	4	Reg#03	36 months	27	Registered nurse
EngCL #23	19	> 5 km	Grade 10	Primary education	8	Reg#04	12 months	29	Registered nurse
EenhCL #04	17	> 5 km	Grade 10	Primary education	7				
ODHC #19	18	2 km	Grade 9	Primary education	8				
OngHC #14	17	3 km	Grade 8	Primary education	6				
EenhCL #29	18	> 5 km	Grade 10	Secondary education	5

**Table 2:** Sub-themes and themes

SUB-THEMES	THEMES
Lack of transport and poor road infrastructures	**Reasons for late starting of ANC**
Lack of transport fare and other related financial matters	
Fear of stigma from community	
Undesirable attitudes from health care providers and peers	
Long distance to the nearest clinic	
Motor vehicles in rare occasions	**Mode of transport to the clinic**
Walking	
Long walking distance	**The distance to the nearest clinics**
No clinics in immediate proximity	
Lack of transport fare and other related financial matters	
Lack of transport and poor road infrastructures	
Long queues and waiting time	**Waiting time**
Shortage of staff	
Fear of disclosing pregnancy due to parents’ reaction	**Challenges affecting access to MCHI**
No freedom for decision making	
Long distances to the nearest clinic	
Lack of transport fare and other related financial matters	
The limited source of information for adolescent girls	
Challenges on disclosing pregnancy	
Fear of stigma from community	
Harsh treatment of adolescent girls by parents and family members	
Undesirable attitudes from pregnant health care provider and adolescent girls	
Poor of availability of MCHI packages or policy guidelines	
Lack of transport and poor road infrastructures	

### Reasons for late starting of antenatal care

#### Lack of transport and poor road infrastructure

Four out of 12 participants and two out of 8 nurses stated that there is limited transport. They further stated that poor road infrastructure to the clinic contributed to transport challenges, resulting in pregnant adolescents walking long distances to the clinics. The scarcity of cars in their areas permeated throughout the different interviews.

*‘There are not many cars. If you get one, it's only those going to work, but that's it. The cars do not pass the road many times. There are times that I walk three hours to the clinic to attend ANC visits if I didn't get transport. I go back at 10 am, because I also wait for the cars at the shopping centre.'* **P2.**

*'When the pregnant adolescent girls come to start ANC visits and we ask them why they are starting late, they always tell us that it is because of transport.'* **RN.**

#### Lack of transport fare and other related financial matters

Three out of 12 participants and 5 out 8 Registered Nurses were in agreement that insufficient financial support from parents and partners was a major challenge limiting pregnant adolescent girls' access to MCHI at the clinics and health centres.

*'The transport might be available but the money is scarce. You do not know where to get money and sometimes the parents do not have jobs nor your partner. Very often you do not have an income and the person who is supposed to help you does not have one either.' P3*.

*'Sometimes they do not have money to come to the clinic, some are staying very far and there is no one to support them financially.'* **EN.**

#### Mode of transport to the clinic

Seven out of 12 participants stated that, the common mode of transport that facilitating them to reach the nearest clinic was mostly by walking. However, long walking distances was mentioned by majority of the pregnant adolescent girls. Those that are able to get cars, it was again attached to lack of financial incentives which hinder them to be dropped at the nearest clinic.

“*The cars used to drive by sometimes, but if you do not have transport money, you cannot hike, so you can just walk on feet, no one will take you with if you do not have* *transport fare”.* **(P9).**

*“I come with a car when coming to attend my ANC visits at the clinic”.* **(P10).**

*“I would go walking. I would just wake up early in the morning, when the sun rises, I am already on my way”. “Yes, when the clinic opens, I am already there after walking 3 hours”.* **(P2).**

### The distance to the nearest clinics

#### Long walking distance

Eight out of 12 participants and 5 out of 8 nurses stated that they were not accessing MCHI, mainly due to long walking distances to the clinics and health centres for ANC visits. One of the pregnant adolescents who was interviewed stated as follows:

*'It is very long! I really do not know how long it is, but the distance is very long, if you go at 8 am, you might reach at 11 am. I would go walking. I would just wake up early in the morning when the sun rises, I am already on my way. Yes, when the clinic opens, I am already there after walking for three hours* **(P1).**

*'They are just far from the health centre and there are no nearby clinics that they can attend to, like Omhedi village, there is no clinic there and it is far from the health centre itself. The women coming from that village coming to attend ANC visits here is very far from the clinic.'* EN**.**

### Waiting time

#### Long queues and waiting time

Nine out of 12 participants stated that they were facing long waiting hours due to long queues, which deterred them from honouring their next appointments and 6 out of 8 nurses also raised similar concerns about the long queues and long waiting hours, leading to pregnant adolescent girls getting bored and at times sneaking away before getting ANC services.

*'It takes me round about four hours to finish when I go for ANC visits. The clinic opens at eight, and I am most of the time second or fourth in the queue because I almost arrive at six o'clock, and by twelve o'clock I am done.'* **P1.**

*'Sometimes the queue is the challenge. Sometimes when the pregnant adolescent girls get here and look at the number of people queued up, they do not join the queue because they are afraid to stay until late, it discourages them too, and some who would like to wait they just end up waiting in the queue till dawn.'* **RN.**

### Challenges affecting access to MCHI

Four out of 12 participants raised their concerns that they could not access the MCHI due to challenges related to the attitudes and behaviour of staff at the clinics and health centres. Unpleasant attitudes and behaviour were not only displayed by staff, but also by their fellow pregnant adolescent peers. These difficulties were further complicated by family dynamics and psychosocial challenges. Moreover, 5 out of 12 nurses were concerned about the undesirable attitudes of the pregnant adolescent girls towards the healthcare staff and vice versa.

#### Undesirable healthcare provider attitudes

Four out of 12 participants raised their concerns pertained to being shouted and scolded at by the nurses. Three out of 8 nurses expressed their views regarding their colleagues' negative attitudes towards pregnant adolescent girls.

*'I remember there was a time where I was experiencing lower abdominal pain. I consulted a nurse about it and she shouted at me, said 'I do not know' but in a harsh voice. I just did not know what she meant.'* ***P1.***

*'Some pregnant adolescent girls will tell you that they went to start with ANC visits and the nurses were talking to them in a shouting manner, meaning the nurses attended to them in a bad way, shouting at them made them feel not happy and uncomfortable.'* **EN.**

#### Undesirable pregnant adolescent girls' attitudes

It was indicated that 6 out of 12 participants were also presenting undesirable behaviour and attitudes, either towards family members or nurses. Three out of 8 nurses raised their concerns that some adolescent girls were concealing their pregnancy and were reluctant to start ANC, for no apparent reason despite family members convincing them to do so as a result it is difficult for them to access relevant MCHI.

*'When I went after three months, I didn't really know what ANC was all about. They just used to insist that I go to the hospital. And I would deny and tell them that I was not pregnant. I was hiding the pregnancy.'* **P1.**

*'On the other hand, adolescents have a high defense mechanism, when their parents try to talk to them they will be answering back and using vague language, to a point where the parents will give up and never mention anything regarding their pregnancy. They go through denial stages where they would not want parents to mention anything about them sleeping out. Insulting parents, these parents do come here with complaints of such, and when we take their blood pressure, it is high.'* ***RN.***

#### No freedom for decision making and fear of disclosing pregnancy due to parents’ reaction

Ten out of 12 participants raised numerous concerns relating to accessing and utilizing MCHI, which mainly revolved around being afraid to go for ANC and to get maternal and child health-related information, given that they would have to first disclose their pregnancies to their parents before starting the ANC. Seven out of 8 nurses also raised their concerns about parents making decisions as to when adolescent girls should start ANC and what to wear at the clinic. Furthermore, six out of 8 nurses expressed their views about the harsh treatment pregnant adolescent girls were receiving from their parents and family members after disclosing their pregnancy. Four out of 12 participants living in the school hostel started their ANC visits late because they did not inform their parents first, despite living in the proximity to the clinic.

*'I was afraid to tell my people at home that I was going to the clinic because I was afraid to be scolded since I am too young to become pregnant.' P4. *

*'They tell us that they were apparently scared to disclose their pregnancy to their parents. They mostly just start with ANC when the parents are aware of the pregnancy, as the parents are the ones that encourage them to start with ANC’.* ***RN.***

#### Fear of stigma from community

Nine out of 12 participants stated that they blame themselves for becoming pregnant at a young age, and which also affected access to MCHI. They also stated that they face stigma from other girls in the community and at school when they fell pregnant. Four out of 8 nurses confirmed the pregnancy-related stigma pregnant adolescent girls face from community members. This category is classified under two sub-categories.

*'I just thought maybe they will beat me up since I became pregnant at a young age.'* ***P1*.**

The second stigma came from peers who would laugh and stare at pregnant adolescents as they pass by.

*'About challenges, is just that there is always that one person that will laugh at you that you are pregnant. It can be your friends that will laugh at you, or tell you that they don't want to hang with you no [any] more since you are now pregnant, or they just laugh when you walk past them. That was what I experienced.'* **P1.**

*'First of all, these children feel embarrassed. They feel embarrassed because sometimes the people from their house do not even know of the pregnancy.'* ***EN*.**

Lack of services was reported as one of the challenges faced by pregnant adolescent girls. Some lacked the requisite knowledge needed to start ANC visits. Some clinics and health centres lacked the necessary services and materials to provide MCHI to the pregnant adolescent girls, as illustrated in the three sub-themes below.

#### The limited source of information for adolescent girls

All participants indicated that information related to maternal and child health is only being accessed from the health facilities. All nurses indicated that no information, education, and communication (IEC) materials are being distributed in the community, either from the clinics or health centres, since they are neither available nor supplied.

*'No, I wasn't aware that I needed to go to the clinic when my periods stopped.'* ***P4*.**

*'What I think makes these girls start with ANC in the second trimester, most people just lack information on the best time to start with ANC. Most of them do not have the information on when to start with ANC. Most of the time when they start and we ask them why they are starting this late they tell us they just did not know when they were supposed to start. There is really just a lack of information among the people.'* ***EN*.**

#### Poor of availability of MCHI packages or policy guidelines

All participants and nurses shared their concerns regarding the lack of policy or guidelines for maternal and child healthcare at the clinics. Nurses only summarized textbook information obtained from training institutions. It was further revealed that each clinic or health centre has its own way of getting information to the pregnant adolescent girls. There are no IEC materials on maternal and child health-related information available to the public.

*'We do not receive any leaflets at all, we use to have leaflets about three years back but these years [at this time] we do not have them at all.'* ***EN*.**

## Discussion

This study aimed to identify challenges facing adolescent girls in accessing and utilizing MCHI during pregnancy. This is consistent with the SDG 3, which emphasizes ensuring universal access to sexual and reproductive healthcare services, which includes family planning, IEC, and the integration of reproductive health into strategies and programmes [27]. This study conforms to the Global Strategy for Women's, Children's and Adolescents' Health (2016–2030). The main findings revealed that accessibility and utilization of MCHI were major challenges, with special reference to long distances to health facilities, necessitating pregnant adolescent girls to walk long hours to the nearest clinics. Another finding was lack of road infrastructure and unavailability of transport which exacerbated accessibility challenges. Insufficient financial support for transport fare further limited access to MCHI services. Family dynamics added to pregnant adolescents' barriers to access and use of information. Parental controls were also key determinants of whether or not pregnant adolescent girls accessed and utilized clinic-based health services. Moreover, pregnant adolescents' fear, guilt, and anticipated parental reaction made it difficult for them to disclose their pregnancies.

Our study results compare well with what is documented in the literature regarding the long travel distances endured by pregnant women to access maternal healthcare services, which, in turn, affect access and utilization of health information [3, 27–31] and lack of transport and poor road infrastructure increased the challenges. This is consistent with the results of a study conducted in Odisha in India, in so far as accessibility and utilization of MCHI, are concerned [32–34]. Additional studies conducted in Nepal Chitwan district also point to the non-availability of transport and lack of road infrastructure as major challenges to pregnant adolescent girls' access and utilization of health information [29]. Consistent with findings from several other studies conducted in sub-Saharan Africa, lack of financial support from family members and partners also affected pregnant women's ability to access and utilize maternal health services [28–32, 35]. Only a few of the adolescent pregnant girls were receiving financial support from their partners or from their parents, but the majority were struggling to reach the clinics and health centres. In addition, a systematic review conducted across sub-Saharan Africa revealed that the positive economic status of adolescent women is associated with the use of maternal healthcare services [34–38]. Studies conducted in America revealed similar findings that pregnant adolescents experience financial hardship during pregnancy [39, 40].

Furthermore, some studies have revealed that poor adolescents are more likely to disengage from social networks, making them less likely to be reached by programmes aimed at improving maternal health service utilization of adolescent mothers [35,41

### Limitations

The following limitations were observed: the study was confined to adolescents presenting to health facilities for ANC, therefore those were not attending ANCs were not included. These were likely to be the most affected people in so far as accessibility and utilization of MCHI is concerned; the study focused on the age group 15 to19 and the perspectives of other young women are missing. In addition the translation of the study from Oshiwambo to English risked the loss or distortion of some meanings during the interpretation process.

## Conclusion

From the pregnant adolescents’ and nurses’ perspectives, long travel hours to reach the nearest clinics, in terms of road infrastructure, non-availability of transport and transport fare were the key barriers to accessibility and utilization of clinic services. Sound justification required for pregnant adolescent girls to be supported with transport fare inadvertently resulted in early and ill-considered pregnancy disclosure, which often culminated in harsh treatment by parents, thereby further limiting access to maternal and child services. Even in instances where these hurdles are surmounted, long queues at the clinic and the alleged cheekiness of staff and other peers deterred them from finalizing the antenatal care, often resulting in these adolescents giving up the process. Therefore, we recommend a holistic approach inclusive of the community leaders, parents, adolescents themselves and other stakeholders. Most importantly, adolescent girls need to be empowered with information on reproductive health matters.

## Supplementary Information

**Additional file 1.**

## Data Availability

All data generated or analyzed during this study are available from the corresponding author on reasonable request.
