# Supplementary material for: Pregnant adolescents and nurses perspectives on accessibility and utilization of maternal and child health information in Ohangwena Region, Namibia
Source: BMC Pregnancy Childbirth. 2022 Apr 5;22:284. doi: 10.1186/s12884-022-04619-w (PMC8985342; doi:10.1186/s12884-022-04619-w)
Supplement: Supplementary file 1 — Additional file 1. [file 12884_2022_4619_MOESM1_ESM.docx]

**The user-provider perspectives towards the accessibility and utilization of maternal and child health information by pregnant adolescents in the Ohangwena Region, Namibia**

**Guiding tool for pregnant adolescent girls**

1. How did you manage to go to the ANC clinic to start ANC as early as possible? /Why didn’t you start ANC early?
2. How do you reach health facility?
3. How far is the distance between your residence and the health facility?
4. How long are you waiting at the ANC clinic before the services is rendered/How long are you waiting in the queue?
5. What challenges are you facing to access maternal and child health information?
6. How did you manage to attend the ANC follow ups as indicated by the nurses?
7. How do you manage to implement what you have been told?
8. Are you going to school?
9. If so how do you manage to attend ANC at the same time that you need to attend classes.
10. What suggestions/recommendations do you suggest that can be implemented to assist young people who are facing challenges?

**Guiding tool for Nurses**

1. How is the ANC attendance of pregnant adolescent girls?
2. Why do you think are some of the reasons pregnant adolescent and young women starting ANC visit very late?
3. How is maternal and child health information being provided to pregnant adolescent and young women?
4. Do you have maternal and child health information package?
5. What challenges are hindering pregnant adolescent girls to access maternal and child health information?
6. How do you involve parents in the care of their pregnant adolescents?
7. Do you need parental consent to provide maternal health care services?
8. What interventions are in place at your health facility to reach young pregnant women with maternal and child health information?
9. How information, education and communication (IEC) is materials which is related to maternal and child health information being distributed to adolescents’ girls in general?
10. How is adolescent friendly health services being implemented at your health facility?
11. How do you assist adolescent and young women who are coming from school?
12. How do adolescent and young women who are far from health facility utilize the maternal health care information/services?
13. How can AGYW be assisted more to utilize maternal health services?
14. What measures are in place to cater for the AYW who cannot be reached with maternal health care information due to long distances from the health facilities?
15. What suggestion/recommendations do you have especially on AYW who are not attending their ANC on time to attract more of them to come and start their ANC (in their first trimester)?
